# Disparities in the Use of Atherectomy and Intravascular Lithotripsy for Percutaneous Coronary Intervention

**DOI:** 10.1016/j.jscai.2025.103615

**Published:** 2025-05-01

**Authors:** Kyu Lee, Priya Roy, Umair Ahmad, Paul S. Chan, Richard J. Gumina, Kevin Kennedy, Vittal Hejjaji, Ali O. Malik

**Affiliations:** a Division of Cardiovascular Medicine, The Ohio State University Wexner Medical Center, Columbus, Ohio; b Division of Cardiovascular Medicine, Saint Luke’s Mid America Heart Institute, Kansas City, Missouri; c Division of Cardiovascular Medicine, University of Missouri Kansas City, Kansas City, Missouri; d Department of Cardiovascular Medicine, Mount Sinai Medical Center, Miami, Florida; e Department of Cardiovascular Medicine, Fairfield Medical Center, Lancaster, Ohio

**Keywords:** atherectomy, calcified coronary lesion, disparities, intravascular lithotripsy

## Abstract

**Background:**

Atherectomy and intravascular lithotripsy (IVL) facilitate percutaneous coronary intervention (PCI) in calcified coronary disease, and use of these technologies is associated with greater luminal gain and superior intervention success. As atherectomy/IVL gain more widespread acceptance, it is important to understand whether their use differs across levels of social deprivation.

**Methods:**

Within the National Cardiovascular Data Registry CathPCI Registry, we identified 310,124 patients who had a PCI for severely calcified lesions between 2018 and 2023. For each patient, we determined their social deprivation index (SDI) based on residential zip codes. The SDI is a composite measure of area-level social deprivation, with higher values correlating to greater deprivation. Hierarchical logistic regression models evaluated the association of SDI with use of atherectomy/IVL.

**Results:**

Mean age was 70.9 ± 10.6 years, 69.4% were men, and 82.3% were of White race. Atherectomy/IVL was used in 33.0% of PCIs in severely calcified arteries. There was an inverse, graded relationship between SDI and atherectomy/IVL use. These differences were only partially attenuated after adjusting for patient and PCI characteristics. Compared with those residing in neighborhoods with the lowest quartile of social deprivation, those in the third and fourth quartiles of social deprivation were 10% (odds ratio, 0.90; 95% CI, 0.88-0.92; *P* < .001) and 8% (odds ratio, 0.92; 95% CI, 0.90-0.94; *P* < .001), respectively, less likely to have atherectomy/IVL used during PCI.

**Conclusions:**

In the United States, greater social deprivation was associated with lower rates of atherectomy/IVL during PCI for severely calcified coronary artery stenoses, highlighting potential disparities in use of these technologies.

## Introduction

Coronary atherectomy and intravascular lithotripsy (IVL) facilitates percutaneous coronary intervention (PCI) in severely calcified lesions.^1^^,^^2^ Although randomized trials have not shown reduction in long-term ischemic events with routine use of coronary atherectomy in severely calcified lesions,^1^^,^^3^ the use of coronary atherectomy/IVL to facilitate PCI in this patient population has been associated with greater luminal gain and superior strategic success.^4^ Current expert consensus guidelines recommend coronary atherectomy/IVL for calcium modification to facilitate angioplasty and stent implantation in severely calcified coronary lesions.^5^^,^^6^

Prior seminal studies aimed at understanding disparities in cardiovascular care have found a strong inverse correlation between cardiovascular risk and socioeconomic strata, with socioeconomically disadvantaged individuals being at an increased risk for the development and progression of coronary artery disease.^7^^,^^8^ Furthermore, studies have demonstrated that socioeconomic status can have a profound impact on utilization of invasive cardiovascular testing, speaking to the effect of income on access to cardiovascular care.^9^

Eliminating disparities in health care is a national priority. As newer technologies to aid PCI such as coronary atherectomy and IVL gain more traction and widespread acceptance, it is important to understand whether differences exist in their application and examine the socioeconomic factors that may contribute to this disparity. Insights from these analyses could guide future efforts to ensure more equitable use of technologies for cardiovascular care in the United States.

## Materials and methods

### Study population

We used data from the National Cardiovascular Data Registry (NCDR) CathPCI and included all patients who underwent PCI for severely calcified lesions from January 2018 to December 2023. Detailed methodology for data collection for the NCDR CathPCI registry has previously been described.^10^ Patients who had severely calcified lesions (identified using NCDR CathPCI data code 8021) were included. We excluded patients who had missing data for race, zip codes, and sites with >20% missing zip codes.

### Study outcome

The primary study outcome was utilization of coronary atherectomy and/or IVL for PCI. This was identified using the NCDR CathPCI data codes, as detailed in Supplemental Table S1. These codes were obtained and verified in consultation with cardiovascular coding specialists at 2 different tertiary care centers.

### Social deprivation index

Social deprivation index (SDI) is a composite measure of area-level deprivation constructed from multiple demographic and household characteristics collected in the American Community Survey, including the following: (1) percentage living in poverty, (2) percentage with <12 years of education, (3) percentage of single-parent households, percentage living in rented housing units, percentage living in an overcrowded housing unit, (4) percentage of households without a car, and (5) percentage of unemployed adults under the age of 65 years.^11^ Detailed methodology for the calculation and validation of SDI has been previously described.^12^ In summation, SDI quantifies the levels of deprivation across small geographic areas and indicates the extent to which a community is socially disadvantaged. The relationship between SDI and the severity of deprivation is direct, positive, and linear; as SDI increases, the severity of deprivation increases, as well. SDIs for our study population using the patient’s residential zip codes were merged to patient level data, and the linked data were provided to us by the NCDR CathPCI registry.

### Statistical plan

We described baseline characteristics and lesion characteristics in our study population. Categorical variables were summarized as frequency (n) and percentage (%). Continuous variables were summarized as mean ± SD for normally distributed variables and median and IQR (25th and 75th percentile) for nonnormally distributed variables. Continuous variables were compared among quartiles of SDI using a linear trend test. Categorical variables were compared among quartiles of SDI using the Mantel–Haenszel trend test.

To assess whether use of atherectomy/IVL for PCI in severely calcified lesions differed by patients’ neighborhood-level social deprivation, we constructed hierarchical logistic regression models (to account for clustering at site level) to evaluate the association between SDI and the use of atherectomy/IVL. First, we assessed the unadjusted association of higher levels of social deprivation (higher SDI score) with use of atherectomy/IVL for PCI in severely calcified coronary lesions. We then adjusted for potential confounders, including patient factors (age, sex, medical comorbidities [hypertension, smoking status, peripheral arterial disease, diabetes mellitus, renal function, prior coronary artery bypass graft surgery, prior PCI, and presence of cardiogenic shock]), lesion-specific factors (lesion length, complexity, bifurcation lesion, and left main lesion), and PCI indication (myocardial infarction vs other) in the model to derive adjusted estimates of effect between SDI and use of atherectomy/IVL during PCI.

All analyses were evaluated at a 2-sided significance level of .05. All analyses were performed with SAS version 9.4 (SAS Institute).

## Results

**Figure 1:**
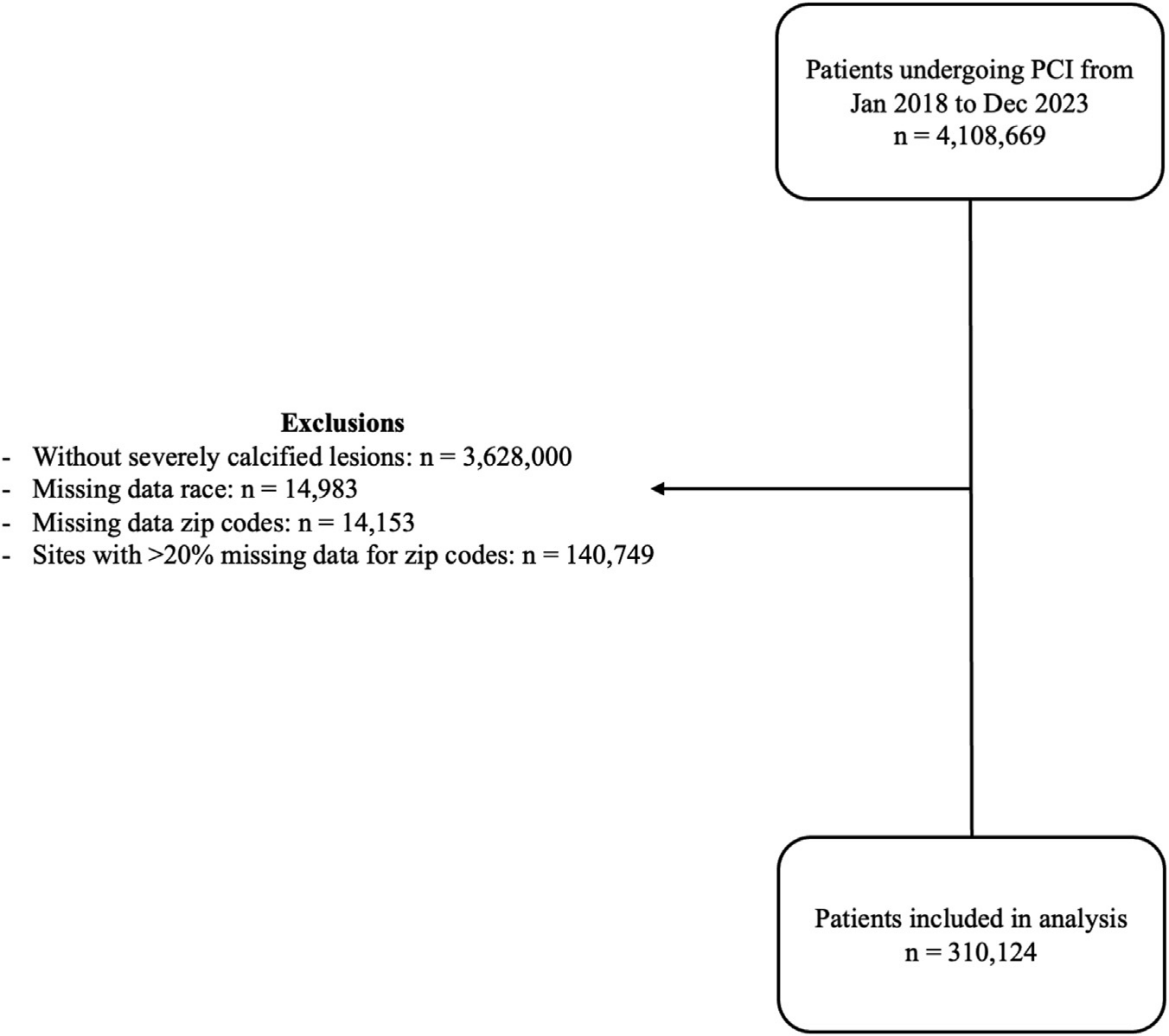
**Derivation of analytical cohort**. PCI, percutaneous coronary intervention.

After excluding patients without severely calcified coronary lesions, missing data on race or zip codes, and sites with >20% missing zip codes, our final study cohort was 310,124 PCI procedures (Figure 1). In our study cohort, the mean age was 70.9 ± 10.6 years, 69.4% of patients were men, and 82.3% were of White race. Overall, the mean SDI was 47.1 ± 27.3. Mean SDI scores in the 4 quartiles (from first quartile to fourth quartile) were 13.2 ± 7.2, 38.0 ± 7.3, 62.6 ± 7.1, and 86.8 ± 6.9, respectively.

Atherectomy and/or IVL was used in 102,356 (33%) of the procedures. Table 1 compares patient demographic characteristics, comorbidities, and PCI indications between quartiles of SDI. Patients in higher quartiles of SDI were younger and less likely to be of White race. There was a higher prevalence of cardiovascular comorbidities (hypertension, smoking, peripheral artery disease, prior coronary revascularization, and worsening renal function) in patients in higher quartiles of SDI. Moreover, higher quartiles of SDI were more likely to present with myocardial infarction or shock and were more likely to not have health insurance.

**Table 1:** Comparison of patient characteristics and comorbidities by quartiles of SDI.

Characteristics	First quartile (SDI 13.2 ± 7.2)	Second quartile (SDI 38.0 ± 7.3)	Third quartile (SDI 62.6 ± 7.1)	Fourth quartile (SDI 86.8 ± 6.9)	*P*-value trend
Demographics					
Age, y	72.1 ± 10.2	71.4 ± 10.5	70.5 ± 10.8	69.1 ± 11.0	<.001
Male sex	61,691 (73.7%)	60,349 (70.2%)	55,218 (67.6%)	38,010 (64.6%)	<.001
White	76,508 (91.4%)	75,938 (88.4%)	67,678 (82.9%)	35,016 (59.5%)	<.001
Black	2145 (2.6%)	3510 (4.1%)	6029 (7.4%)	12,194 (20.7%)	<.001
Hispanic	1867 (2.2%)	3063 (3.6%)	5047 (6.2%)	8508 (14.5%)	<.001
Other minorities	3174 (3.8%)	3408 (4.0%)	2897 (3.5%)	3142 (5.3%)	<.001
Comorbidities					
Hypertension	74,741 (89.3%)	77,511 (90.2%)	74,056 (90.7%)	54,116 (91.9%)	<.001
Smoker	47,287 (56.5%)	51,360 (59.8%)	49,353 (60.4%)	34,593 (58.8%)	<.001
PAD	12,817 (15.3%)	14,099 (16.4%)	14,401 (17.6%)	11,450 (19.5%)	<.001
Prior MI	24,152 (28.9%)	25,977 (30.2%)	25,125 (30.8%)	18,033 (30.6%)	<.001
Prior PCI	34,961 (41.8%)	36,260 (42.2%)	34,227 (41.9%)	24,140 (41.0%)	.007
Prior CABG	14,206 (17.0%)	14,990 (17.4%)	14,132 (17.3%)	9556 (16.2%)	.002
Diabetes mellitus	36,082 (43.1%)	39,739 (46.3%)	40,159 (49.2%)	31,846 (54.1%)	<.001
eGFR, mL/min/1.73 m^2^	71.7 ± 29.5	70.9 ± 30.4	70.2 ± 31.4	68.6 ± 34.7	<.001
Current dialysis	3523 (4.2%)	4448 (5.2%)	5086 (6.2%)	6195 (10.5%)	<.001
Presentation for PCI					
STEMI	7731 (9.2%)	8505 (9.9%)	8567 (10.5%)	6261 (10.6%)	<.001
Non-STEMI	27,515 (32.9%)	30,208 (35.2%)	29,573 (36.2%)	22,573 (38.4%)	<.001
Shock	2313 (2.8%)	2635 (3.1%)	2760 (3.4%)	2224 (3.8%)	<.001
Socioeconomic factors					
Private insurance	19,805 (23.7%)	21,700 (25.3%)	21,945 (26.9%)	15,037 (25.5%)	<.001
Medicaid	4377 (5.2%)	7087 (8.2%)	10,021 (12.3%)	11,849 (20.1%)	<.001
Medicare/military	57,789 (69.0%)	54,963 (64.0%)	47,273 (57.9%)	29,685 (50.4%)	<.001
No insurance	1723 (2.1%)	2169 (2.5%)	2412 (3.0%)	2289 (3.9%)	<.001

Values are mean ± SD or n (%).

CABG, coronary artery bypass graft surgery; eGFR, estimated glomerular filtration rate; MI, myocardial infarction; PAD, peripheral artery disease; PCI, percutaneous coronary intervention; SDI, social deprivation index; STEMI, ST-segment elevation myocardial infarction.

Table 2 compares lesion characteristics across quartiles of SDI. There were no significant trends in lesion length and those undergoing left main PCI among the quartiles of SDI. However, patients in higher quartiles of SDI had a lower proportion of complex lesions (American College of Cardiology group C) and bifurcation lesions. There was a higher proportion of PCIs for chronic total occlusions in patients in higher quartiles of SDI.

**Table 2:** Comparison of lesion characteristics by quartiles of SDI.

Characteristic	First quartile	Second quartile	Third quartile	Fourth quartile	*P*-value trend
Lesion length, mm	42.4 ± 30.1	42.5 ± 30.5	41.9 ± 30.3	42.5 ± 31.0	.927
High lesion complexity	69,469 (83.0%)	70,999 (82.6%)	66,834 (81.9%)	48,285 (82.0%)	<.001
Chronic total occlusion	6049 (7.2%)	6193 (7.2%)	5712 (7.0%)	4520 (7.7%)	.038
Bifurcation lesions	18,418 (22.0%)	18,902 (22.0%)	17,494 (21.4%)	12,307 (20.9%)	<.001
Left main PCI	8423 (10.1%)	8717 (10.1%)	8186 (10.0%)	5893 (10.0%)	.594

Values are mean ± SD or n (%).

PCI, percutaneous coronary intervention; SDI, social deprivation index.

In unadjusted analysis, there was an inverse, graded relationship between SDI and use of atherectomy/IVL for PCI. Patients in the highest quartile of SDI were 15% less likely to have atherectomy/IVL used for PCI in severely calcified coronary lesions. After adjusting for patient age, cardiovascular comorbidities, lesion-specific characteristics, and PCI indication, there was some attenuation of these differences. However, compared with patients in the lowest quartile of SDI, those in the third and fourth quartile of SDI were 10% and 8% less likely to have atherectomy/IVL used during their PCIs (Table 3, Central Illustration).

**Table 3:** Likelihood for use of atherectomy/intravascular lithotripsy for percutaneous coronary intervention in calcified coronary lesions.

SDI quartile	Model 1 (unadjusted)		Model 2 (adjusted)^a^	
	Odds ratio (95% CI)	*P* value	Odds ratio (95% CI)	*P* value
Second vs first quartile	0.93 (0.92-0.95)	<.0001	0.96 (0.94-0.98)	.0002
Third vs first quartile	0.85 (0.83-0.87)	<.0001	0.90 (0.88-0.92)	<.0001
Fourth vs first quartile	0.85 (0.83-0.87)	<.0001	0.92 (0.90-0.94)	<.0001

SDI, social deprivation index.

a This model is adjusted for age, sex, medical comorbidities (renal function, prior coronary artery bypass graft surgery, prior percutaneous coronary intervention, presence of cardiogenic shock), lesion-specific factors (lesion length, complexity, vein graft lesion, previously treated lesion), and percutaneous coronary intervention indication (myocardial infarction vs other).

**Central Illustration:**
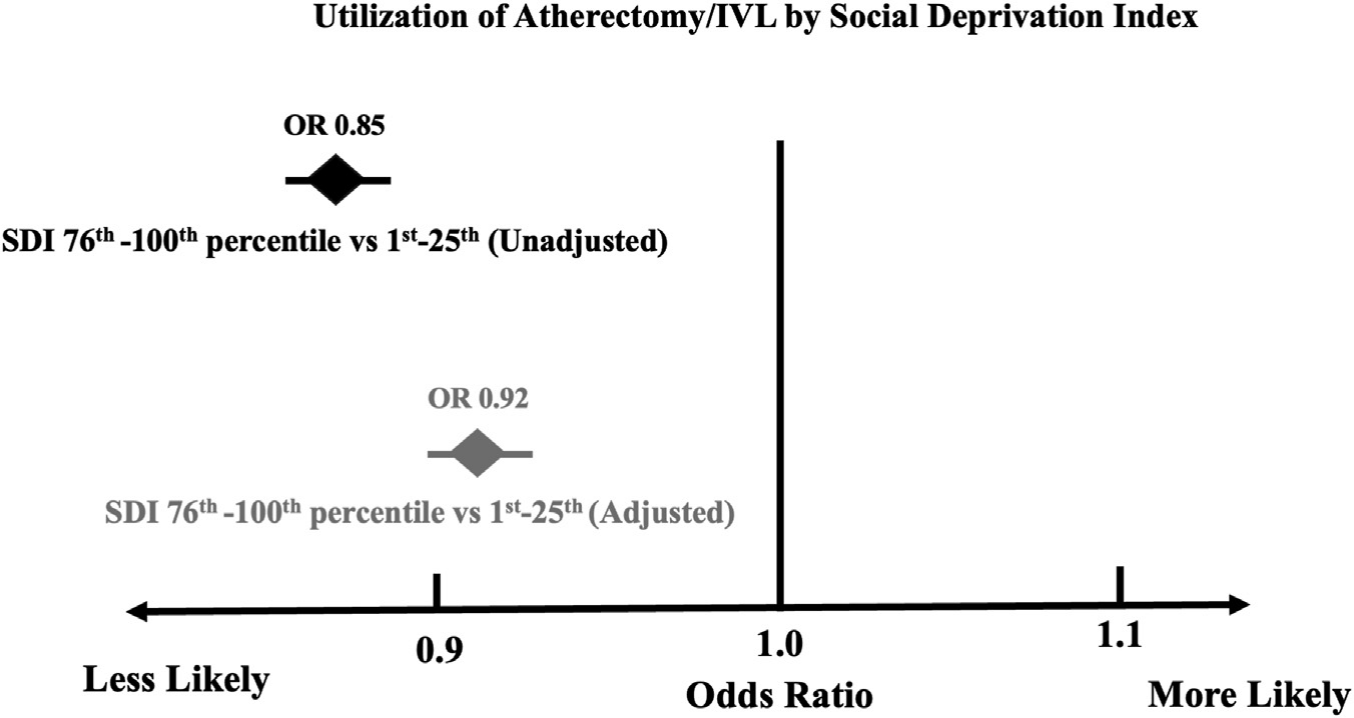
**Odds ratio (OR) for use of atherectomy/intravascular lithotripsy (IVL) for percutaneous coronary intervention in severely calcified coronary lesions.** Unadjusted (top panel). Adjusted for patient/lesion factors and percutaneous coronary intervention indication (bottom panel). SDI, social deprivation index.

## Discussion

Expert consensus guidelines recommend atherectomy/IVL for severely calcified coronary stenoses to facilitate angioplasty and stent implantation.^5^^,^^6^ As more medical centers and providers have taken up this technology, the use of atherectomy/IVL for PCI in the United States has steadily increased.^13^^,^^14^ Our study aimed to assess whether use of these therapies differed by patients’ neighborhood-level social deprivation in the United States within a national PCI registry. We found that patients with higher social deprivation had a lower likelihood of receiving atherectomy/IVL for PCI in severely calcified coronary disease.

Studies have shown that cardiovascular outcomes and care may differ by patients’ socioeconomic status.^15^^,^^16^ Income level, education, employment status, and environmental factors have been shown to be associated with the development and progression of cardiovascular disease, and socioeconomic status may contribute to cardiovascular risk to a degree that is equivalent to traditional cardiovascular risk factors.^17^, ^18^, ^19^ Persons with low socioeconomic status have also been shown to have disproportionately higher levels of stress, depression, and anxiety, which in turn are known to increase the risk of mortality from coronary heart disease.^20^ With respect to environmental factors, population analyses have shown that living in disadvantaged areas was linked to a higher incidence of coronary heart disease, even after controlling for income, education, and employment status.^21^ Although these studies have reported on differential rates of disease incidence and severity, they are often outside the locus of direct control of health care providers. In contrast, our study focused on differential treatment during PCI, which is under the locus of control of health care providers.

Social deprivation index is a composite measure of area-level deprivation constructed from multiple demographic and household characteristics, which includes metrics of poverty, education, and unemployment.^11^ SDI quantifies the levels of deprivation across small geographic areas and indicates the extent to which a community is socially disadvantaged. Our study found that individuals with a higher level of social deprivation were less likely to receive atherectomy/IVL in PCI of severely calcified coronary lesions, even after adjusting for patient demographic characteristics, comorbidities, lesion characteristics, and clinical presentation. Moreover, we constructed hierarchical models to additionally control for the hospital site at which patients received care. In essence, we found that after controlling for the hospital site effect, patients residing in neighborhoods with higher social deprivation were less likely to receive potentially important adjuncts during PCI for severely calcified coronary stenoses, highlighting a potential disparity in use of these technologies.

Atherectomy and IVL are associated with both direct device-related costs as well as a number of indirect costs (eg, increased procedure times, procedure-room cost, and device maintenance), which together can amount to several thousands of dollars in added expenses over traditional angioplasty. Therefore, system expenses and compensation can be critical factors in deciding whether a patient receives atherectomy/IVL during PCI of their calcified coronary lesions. Further compounding this issue is the recent implementation of new coding updates with respect to billing that increase compensation to health systems that use IVL.^22^ While such policies could increase the usage of IVL across PCI centers in the country, there may be a need to have systems in place to allow all patients access to these technologies, regardless of income or other socioeconomic factors.

There are several limitations to our study. First, although this study cohort was taken from a national PCI registry, it may not be generalizable to the entire population in the United States. White patients were disproportionately represented in our study (82.3%), compared with 58.4% nationally.^23^ Second, several sites in the registry had very high rates of missing data for patient zip codes. We decided to exclude sites from our analysis that had missing zip code data for greater than 20% of cases; while excluding these sites decreased the cohort size, this was done to maximize the proportion of patients with available SDI information. Finally, our analytical cohort included patients who underwent intervention from January 2018 to December 2023. Recent randomized clinical trials have since updated the evidence base regarding the benefit of coronary atherectomy in patients with severely calcified lesions.^24^ Future studies evaluating the use of atherectomy/IVL for PCI may provide additional insights as to whether these recent guideline updates have reduced differential use of atherectomy/IVL by patients’ SDI.

## Conclusions

Within a large US registry of patients who underwent PCI for severely calcified coronary disease, we found that patients with higher degrees of social deprivation were less likely to receive therapies for calcium modification to facilitate PCI. These results highlight potential socioeconomic disparities in the usage of atherectomy/IVL across the country.
