# Supplementary material for: Disparities in the Use of Atherectomy and Intravascular Lithotripsy for Percutaneous Coronary Intervention
Source: J Soc Cardiovasc Angiogr Interv. 2025 May 1;4(7):103615. doi: 10.1016/j.jscai.2025.103615 (PMC12418460; doi:10.1016/j.jscai.2025.103615)
Supplement: Supplementary Table 1 [file mmc1.pdf]

SUPPLEMENTARY MATERIALS

Supplemental Table S1. ICD codes for IVL/atherectomy.

**NCDR Cath PCI Codes**	
Data Entry	NCDR Cath PCI Codes
Calcified Lesion	8021
FLEXI-CUT Atherectomy	3511
Trerotola PTD Rotator Drive Unit	3531
Rotablator rotational atherectomy system	3575
Silver Hawk Plaque Excision System	3590
DIAMONDBACK 360	3744
RotaLink Plus Rotational Atherectomy System	3792
RotaLink Plus Rotational Atherectomy System	3803
Atherectomy	3843
ROTAPRO Rotational Atherectomy System	4152
Intravascular Lithotripsy (IVL) System	4509
Shockwave Intravascular Lithotripsy (IVL) System	4923
Shockwave C2+ Coronary IVL	9102
